# Actuarial survival of a large Canadian cohort of preterm infants

**DOI:** 10.1186/1471-2431-5-40

**Published:** 2005-11-09

**Authors:** Huw P Jones, Stella Karuri, Catherine MG Cronin, Arne Ohlsson, Abraham Peliowski, Anne Synnes, Shoo K Lee, The Canadian Neonatal Network

**Affiliations:** 1 Department of Pediatrics, St Mary's Hospital, Portsmouth, UK; 2 Canadian Neonatal Network Coordinating Centre, Edmonton, AB, Canada; 3 Department of Pediatrics, University of Manitoba, Winnipeg, MB, Canada; 4 Department of Pediatrics, University of Toronto, Toronto, ON, Canada; 5 Department of Pediatrics, University of Alberta, Edmonton, AB, Canada; 6 Department of Pediatrics, University of British Columbia, Vancouver, BC, Canada

## Abstract

**Background:**

The increased survival of preterm and very low birth weight infants in recent years has been well documented but continued surveillance is required in order to monitor the effects of new therapeutic interventions. Gestation and birth weight specific survival rates most accurately reflect the outcome of perinatal care. Our aims were to determine survival to discharge for a large Canadian cohort of preterm infants admitted to the neonatal intensive care unit (NICU), and to examine the effect of gender on survival and the effect of increasing postnatal age on predicted survival.

**Methods:**

Outcomes for all 19,507 infants admitted to 17 NICUs throughout Canada between January 1996 and October 1997 were collected prospectively. Babies with congenital anomalies were excluded from the study population. Gestation and birth weight specific survival for all infants with birth weight <1,500 g (n = 3419) or gestation ≤30 weeks (n = 3119) were recorded. Actuarial survival curves were constructed to show changes in expected survival with increasing postnatal age.

**Results:**

Survival to discharge at 24 weeks gestation was 54%, compared to 82% at 26 weeks and 95% at 30 weeks. In infants with birth weights 600–699, survival to discharge was 62%, compared to 79% at 700–799 g and 96% at 1,000–1,099 g. In infants born at 24 weeks gestational age, survival was higher in females but there were no significant gender differences above 24 weeks gestation. Actuarial analysis showed that risk of death was highest in the first 5 days. For infants born at 24 weeks gestation, estimated survival probability to 48 hours, 7 days and 4 weeks were 88 (CI 84,92)%, 70 (CI 64, 76)% and 60 (CI 53,66)% respectively. For smaller birth weights, female survival probabilities were higher than males for the first 40 days of life.

**Conclusion:**

Actuarial analysis provides useful information when counseling parents and highlights the importance of frequently revising the prediction for long term survival particularly after the first few days of life.

## Background

The improvement in survival rates for preterm and very low birth weight infants has been well documented during the last 20 years [1-3]. More recently, a marked reduction in mortality rates has been reported following the introduction and widespread use of antenatal steroids and exogenous surfactant [4-6]. Despite initial concerns, studies of later neurodevelopmental outcome have not shown an increase in major disability rates as a result of improved survival [7-9]. As our understanding of disease processes increases and new therapies continue to be developed, continued surveillance of up to date outcome data is essential in order to monitor the effectiveness of current practice.

Early reports of survival rates for high risk infants were based on birth weight alone, as assessment of gestational age was relatively imprecise prior to the introduction of routine first trimester ultrasound scans. Subsequently, survival data based on gestational age has become more widely reported [10,11]. This information is more useful for obstetric decision-making in the prenatal period and there is increasing evidence that mortality and later morbidity in high risk infants relates more closely to gestation than to birth weight [10,12]. Indeed a combination of both variables may give an even more accurate prediction of outcome once the infant is born [13].

Of further concern is that survival data in many published studies is derived from single tertiary units or collaborations of such centres which are not geographically based [1,14]. This increases the likelihood of sample bias and may not give a true reflection of survival expectations for the population as a whole. The effects of gender and multiple births on survival have also been of interest in previous studies, many showing higher survival rates for females and/or singletons [11,15-19]. This additional information may enhance our prediction of survival based on gestation or birth weight alone. More recently, attention has focused on the timing of infant death, specifically utilising actuarial survival analysis to predict future life expectancy from a given age [20-23]. This provides useful additional information when making decisions regarding ongoing management in the neonatal intensive care unit (NICU).

Our aims were to determine survival to discharge for a large Canadian cohort of high-risk infants (<1500 g or ≤30 weeks) representing admissions to 17 NICUs with 75% of tertiary level NICU beds in Canada, and to examine the effects of gestation, birth weight, gender and multiple birth on survival. Utilizing actuarial survival analysis, the effect of increasing postnatal age on expected survival to discharge was investigated.

## Methods

### Study population

The Canadian Neonatal Network comprised 17 tertiary NICUs across Canada in 1996 [24]. It was funded by the Medical Research Council of Canada and other institutions (see acknowledgements) in 1996 to facilitate neonatal research by creating a national neonatal-perinatal database. The Canadian population in 1996 was about 30 million with approximately 357,000 births annually [25]. This study included all infants with a birth weight <1500 grams or gestational age ≤30 weeks who were admitted to the Canadian Neonatal Network during a 22-month period between January 1996 and October 1997. Infants with congenital anomalies were excluded because they have different mortality and morbidity risks [26].

### Data collection

Prospective data were collected locally by trained research assistants and transmitted electronically to the Canadian Neonatal Network Coordinating Centre for verification and analysis. Collected data included demographic variables, obstetric information, neonatal illness severity (Score for Neonatal Acute Physiology, Version II [SNAP-II]) [27], therapeutic intensity (Neonatal Therapeutic Intensity Scoring System [NTISS]) [28] and selected outcomes and resource use.

### Definition of study variables

Study variables were defined according to the Canadian Neonatal Network SNAP Project Abstractor Manual [29]. An admission was defined as a stay in the NICU for at least 24 hours or death/transfer to another unit within 24 hours. *Gestational age *was defined as the best obstetric estimate based on early prenatal ultrasound, obstetric examination and obstetric history, unless the post-natal pediatric estimate of gestation differed from the obstetric estimate by more than two weeks. In that case, the pediatric estimate of gestational age was used instead. An infant was defined as *small-for-gestational age *(SGA) if the birth weight was less than the 10^th ^percentile for gestational age according to the growth charts established by Arbuckle [30] in 1989 for the Canadian population. *SNAP-II *[27] is a neonatal illness severity score calculated from 6 empirically weighted physiologic measurements made during the first 12 hours of admission to the NICU. *NTISS *[28] is a score of neonatal therapeutic intensity calculated from a checklist of 63 NICU therapies used in a 24 hour period, weighted according to invasiveness and cost. *Chronic lung disease *was defined as oxygen dependency at 36 weeks corrected GA for an infant who was born at ≤32 weeks gestation [31]. *Intraventricular hemorrhage *(IVH) was defined according to the criteria of Papile [32] from head ultrasound performed before 14 days of life. *Necrotizing enterocolitis *(NEC) was defined according to Bell's criteria (stage 2 or higher) [33] and was classified as medical (clinical symptoms and signs plus evidence of pneumatosis on abdominal X'ray) or surgical (histological evidence of NEC on surgical specimen of intestine). *Retinopathy of prematurity *(ROP) was defined according to the International Classification for Retinopathy of Prematurity [34] and the Reese Classification of cicatrical disease [35]. *Nosocomial infection *was defined using blood and cerebrospinal fluid culture results according to Freeman's criteria [36]. *Patent ductus arteriosus *was defined as clinical diagnosis plus treatment with indomethacin or surgical ligation or both. *Seizures *were defined as clinically significant episodes witnessed by a nurse or physician and for which anti-convulsant treatment was given. *Congenital anomalies *were classified according to the WHO International Classification of Diseases, 9 th Revision (ICD-9) [37].

### Data analysis

Data analysis was carried out on two populations – preterm babies with gestation age ≤30 weeks and Very Low Birth Weight (VLBW) babies with birth weight <1,500 grams. A two sample t-test was used to separately test the effect of gender and multiple births on population demographics, adverse outcome and resource use. Similarly a two sample t-test was used to test the effect of gender on survival proportions for each week of gestational age and each 100 grams birth weight range. For the actuarial analysis, the event of interest was death and the time-to-event was the number of days survived. Babies that survived to discharge had their length of stay included as right censored observations. The actuarial survival estimator which estimates the probability of survival to and beyond a set number of days was calculated in daily intervals for the first 60 days of life. The effect of gender, gestation age and birth weight on survival were studied by obtaining actuarial survival estimators for each category. The probability of survival to discharge, for an infant who has survived to a given day in the NICU, was calculated and graphed by gestational age and birth weight category.

## Results

Between January 8, 1996 and October 31, 1997, 19,507 infants were admitted to participating NICUs. Excluding those with congenital anomalies, there were 3,119 infants ≤30 weeks gestational age and 3,409 infants with birth weight <1,500 grams. Characteristics of the two groups are shown in Table 1. Of note, for infants <1,500 grams birth weight, 52% were born by caesarean section, 21% were outborn and 68% received antenatal steroids.

**Table 1:** Characteristics of preterm and very low birth weight (VLBW) infants in the study cohort

	**Preterm (≤30 weeks)**	**VLBW (<1500 g)**
	n = 3119	n = 3409

Males (%)	56	53
Multiple births (%)	27	29
Cesarean section (%)	46	51
Antenatal steroids (%)	72	70
Outborn (%)	22	21
Small for gestational age 3 rd percentile (%)	14.7	26.1
Mean birth weight (grams)	1071	1041
Mean gestational age (weeks)	27	28

### Survival to NICU discharge

**Figure 1:**
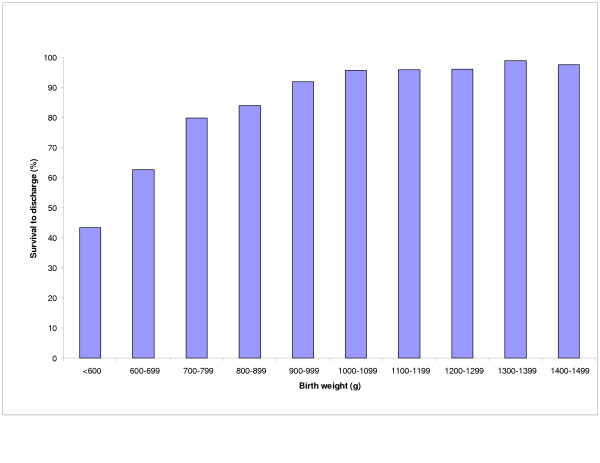
Birth weight specific survival.

**Figure 2:**
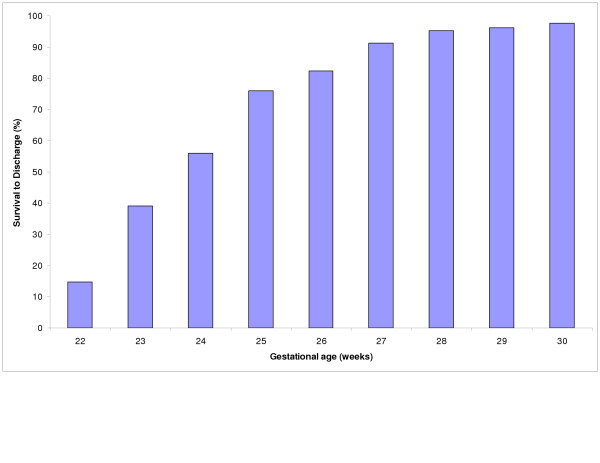
Gestational age specific survival.

Survival to discharge increased from 45% for infants weighing <600 g at birth to over 95% for those with birth weight >1200 g (Fig 1). There was a similar increase for gestational age groups with survival increasing from about 14% at 22 weeks to over 93% at 28 weeks and above (Fig 2).

### Effect of gender on survival and morbidity

**Figure 3:**
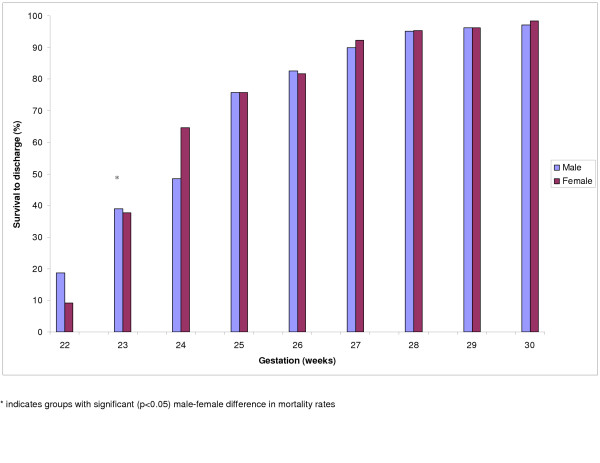
Male/Female survival by gestational age.

**Figure 4:**
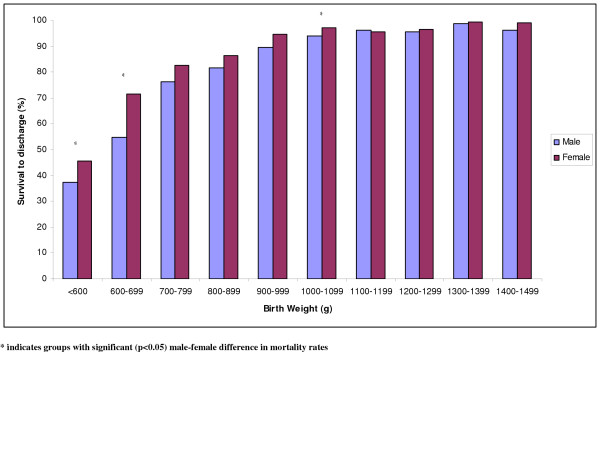
Male/Female survival by birth weight.

When analysed by gestational age groups, survival for male and female infants was not significantly different except for those born at 24 weeks gestation in whom survival for females was 17 (3, 29)% higher (Fig 3). When analysed according to birth weight groups, survival for females in the 600–699 g group was 7 (6,28)% significantly higher than for males (Fig 4). However, within many birth weight groups, females were significantly more mature than males and would therefore be expected to have higher survival as a consequence of gestational age (Tables 2 and 3). In contrast to survival, female infants had significantly (p < 0.05) lower incidence of chronic lung disease and severe intraventricular hemorrhage than male infants (Table 4). The high incidence of SGA among infants born at <1,500 g reflects the inherent tendency to select SGA infants when birth weight criteria is used to categorise infants instead of gestational age.

**Table 2:** Comparison of mean gestational age (GA) between male and female infants in each birth weight group (VLBW)

		**Mean GA (weeks)**		
**Birth Weight (g)**		**Male**	**Female**	**p-value**
	n			
<600	203	24.2	24.4	ns
600–699	303	24.7	25.5	<0.05
700–799	318	25.5	26.0	<0.05
800–899	344	26.3	27.1	<0.05
900–999	297	27.2	27.4	ns
1000–1099	375	28.2	28.6	ns
1100–1199	368	28.8	29.3	<0.05
1200–1299	381	29.4	29.9	<0.05
1300–1399	372	29.7	30.9	<0.05
1400–1499	448	30.7	31.0	ns

**Table 3:** Comparison of mean birth weight between male and female infants in each GA group (Preterm)

		**Weight (grams)**		
**GA (weeks)**		**Male**	**Female**	**p-value**
	n			
23	105	625	576	<0.05
34	234	699	679	ns
25	329	765	755	ns
26	374	897	813	<0.05
27	388	1005	943	<0.05
28	466	1155	1102	ns
29	579	1311	1236	<0.05
30	612	1485	1382	<0.05

**Table 4:** Male/Female characteristics of preterm (≤30 weeks gestation) and very low birth weight Infant (< 1,500 g) groups

	**Preterm (≤30 weeks gestation)**		**Very low birth weight (< 1,500 g)**	
	**Male **n = 1741 (56%)	**Female **n = 1376 (44%)	**Male **n = 1797 (53%)	**Female **n = 1601 (47%)

**Demographics**				
Mean birth weight (grams)	1102	1032*	1050	1031*
Mean gestational age (weeks)	27.4	27.4	27.9	28.3*
Small for gestational age (%)	13.6	16.1*	24.4	28.2*
5-minute Apgar score (mean)	7.2	7.3	7.2	7.4*
Outborn (%)	21.4	21.7	21.4	19.9
Antenatal steroids (%)	69.3	71.3	66.2	69.4*
SNAP-II (mean)	26.6	27.2	26.5	24.8*
				
**Outcomes**				
Survival (%)	85.2	86.9	85.6	88.9*
Necrotizing enterocolitis (%)	6.5	7.0	6.6	6.3
Patent ductus arteriosus (%)	28.0	32.3*	27.3	28.8
Seizures (%)	4.6	4.9	4.5	4.2
Chronic lung disease (%)	25.1	21.7*	25.7	18.9*
Primary infection (%)	1.8	2.0	1.6	1.6
Nosocomial infection (%)	21.2	22.1	21.9	20.9
Intraventricular hemorrhage (≥grade 3) (%)	9.6	7.5*	9.1	6.4*
Retinopathy of prematurity (≥stage 3) (%)	10.9	10.8	10.5	9.7
				
**Resource use**				
NTISS (mean)	17.5	17.1	17.1	16.0*
Assisted ventilation (%)	88.7	86.7	83.9	79.6*
Number of ventilated days (mean)	18.0	17.1	17.5	14.9*
Surgery (%)	21.2	14.5*	21.6	12.9*
Length of NICU stay (mean) (days)	48.1	48.9	47.6	45.7

* significant (p < 0.05) difference between male and female infants

### Multiple births and antenatal steroids

Of the 3119 infants born ≤30 weeks, 2,277 (73%) were singleton deliveries and 841 (27%) were the products of multiple gestation pregnancies (Table 5). There was no significant difference in survival, illness severity or resource use between the two groups. Antenatal steroid use was 7(4,11)% higher in multiple gestation for VLBW babies, and delivery by cesarean section was 9(5,13)% more likely in multiple gestation pregnancies for VLBW. Antenatal steroid use was 7(3,11)% higher in multiple gestation for preterm babies, and delivery by cesarean section was 9(6,13)% more likely in multiple gestation pregnancies for preterm. A full or partial course of antenatal steroids was given to 70% of infants ≤30 weeks gestation and to 68% of infants between <1,500 g birth weight. The use of antenatal steroids was lower in the most preterm infants ≤24 weeks gestation.

**Table 5:** Characteristics of singleton and multiple birth preterm infants

	**Preterm (≤30 weeks gestation)**		**Very low birth weight (< 1,500 g)**	
	**Singleton **n = 2277 (73%)	**Multiple **n = 841 (27%)	**Singleton **n = 2424 (71%)	**Multiple **n = 975 (29%)

**Demographics**				
Mean birth weight (grams)	1071	1072	1031	1066
Mean gestational age (weeks)	27.3	27.6*	27.9	28.4*
Small for gestational age (%)	15.0	13.8	26.3	25.7
5-minute Apgar score (mean)	7.2	7.4*	7.2	7.5*
Outborn (%)	22.5	18.9*	21.9	17.6*
Antenatal Steroids (%)	68.2	75.4*	65.7	73.2*
SNAP-II (mean)	27.1	26.2	26.3	24.1*
				
**Outcomes**				
Survival (%)	86.1	85.7	87.1	87.4
Necrotizing enterocolitis (%)	6.9	6.3	6.8	5.6
Patent ductus arteriosus (%)	29.6	30.9	28.1	28.0
Seizures (%)	5.3	3.2*	4.8	3.2*
Chronic lung disease (%)	24.4	21.7	23.4	20.2
Primary infection (%)	2.2	0.8*	1.9	0.9*
Nosocomial infection (%)	22.1	21.6	22.0	20.0
Intraventricular hemorrhage (≥grade 3) (%)	9.2	7.3	8.4	6.5
Retinopathy of prematurity (≥stage 3) (%)	12.1	7.4*	11.3	7.2*
				
**Resource use**				
Caesarean section (%)	43.0	52.4*	48.7	57.7*
NTISS (mean)	17.3	17.3	16.8	16.1*
Assisted ventilation (%)	87.6	88.6	82.8	79.6*
Number of ventilated days (mean)	18.2	15.9*	17.1	14.1*
Surgery (%)	18.8	17.1	18.4	15.3*
Length of NICU stay (mean) (days)	48.8	47.6	47.9	43.9*

* denotes p < 0.05 level of significance

### Actuarial survival

**Figure 5:**
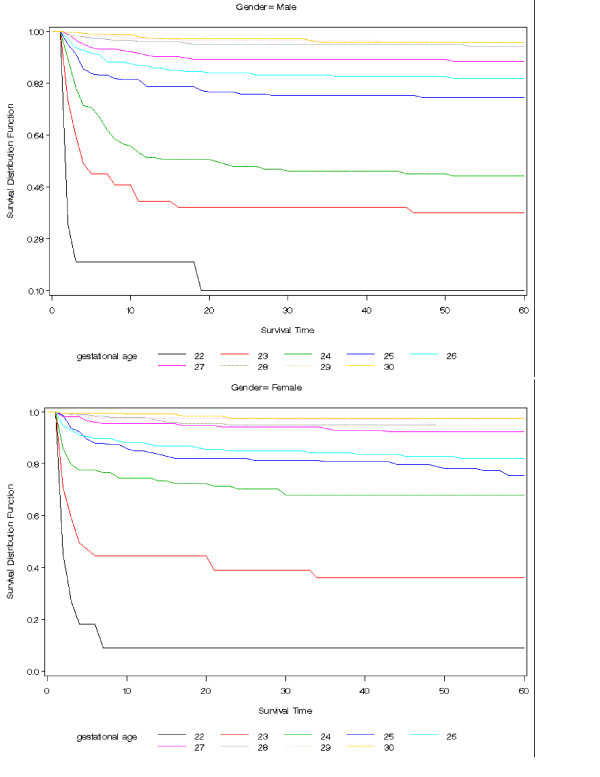
Actuarial survival curves stratified by gestation (weeks), by gender.

**Figure 6:**
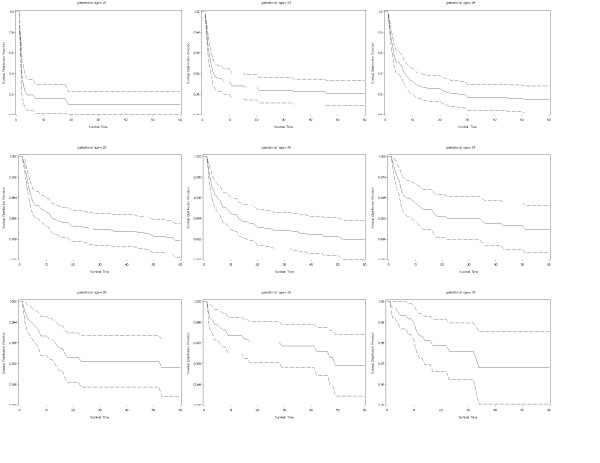
Actuarial survival curves by gestational age, with 95% CI.

**Figure 7:**
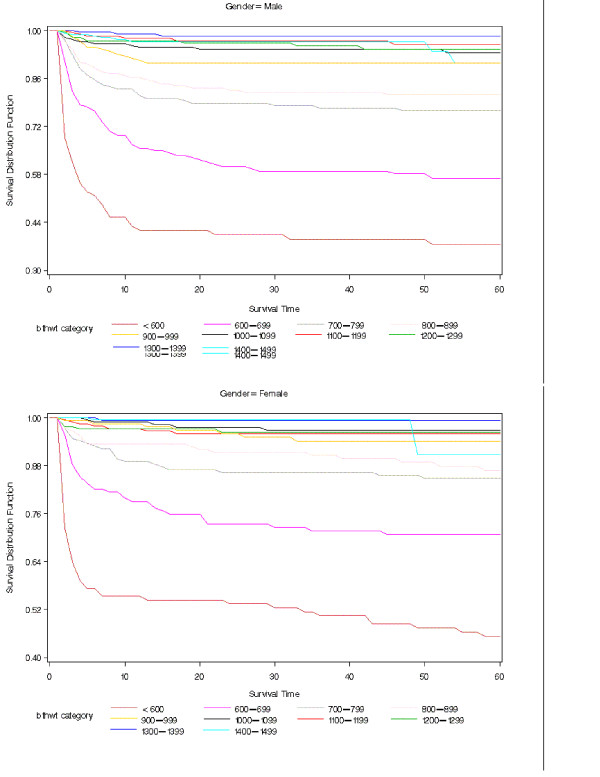
Actuarial survival curves stratified by birth weight category; by Gender.

**Figure 8:**
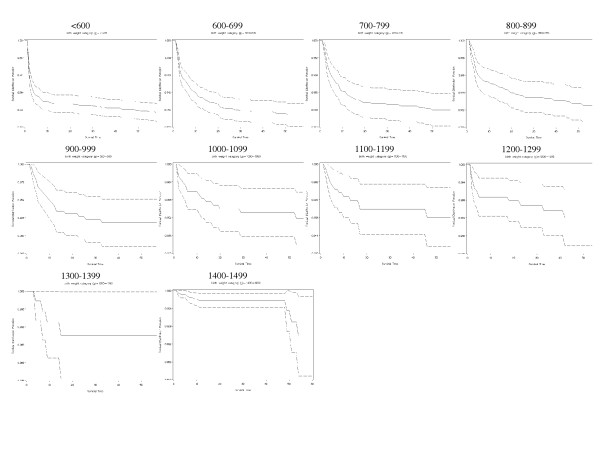
Actuarial survival curves by birth weight category.

**Figure 9:**
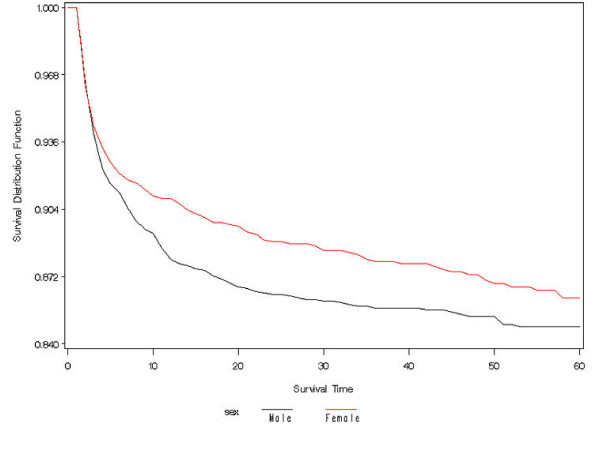
Actuarial survival curves by sex.

**Figure 10:**
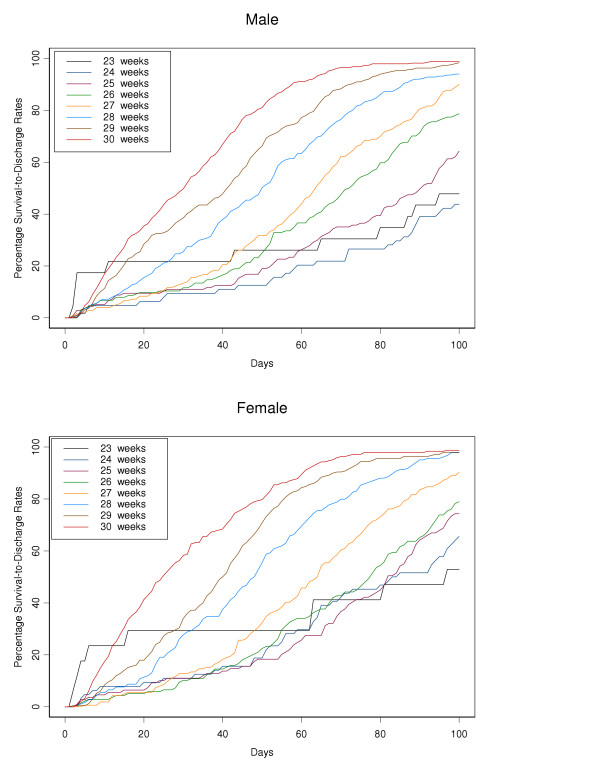
Probability of survival to discharge (y-axis), for male and female infants surviving to a given day in the NICU (x-axis), stratified by gestation age.

**Figure 11:**
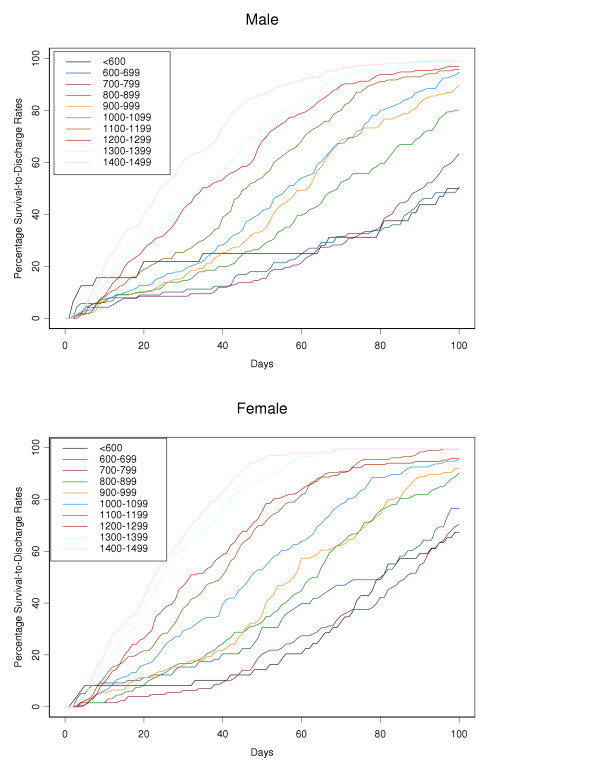
Probability of survival to discharge (y-axis), for male and female infants surviving to a given day in the NICU (x-axis), stratified by birth weight (g).

Actuarial survival curves are given in Figures 5, 6, 7, 8, 9 for the first 60 days. Points on the curves give *S(t)*; the estimated probability of a baby surviving to at least *t *days. Babies that were discharged from the NICU can not afford to be ignored as these were most likely to be the longer lived observations. Babies that were discharged from the NICU were therefore included as censored observations in the analysis. By comparing the slope of the curve (which gives the instantaneous risk of death), the highest risk of death is within the first 6 days. Risk of death is higher for smaller babies and babies with lower GA. Risk of death also decreases with time. Figure 9 shows that actuarial survival is higher among females than males during the first 40 days, and narrows after that. Figures 10 and 11 give the probability of survival to discharge for an infant who has survived to a given day in the NICU, stratified by gestational age and birth weight respectively.

## Discussion

Since our study is based on a large, geographically defined Canadian cohort, it has greater relevance than those from single units and provides a more realistic picture of neonatal outcomes. In addition, our study cohort was derived from large regional centres with comparable levels of care conforming to current North American standards for neonatal intensive care. Our results are similar to those from previous publications on NICU outcomes, such as that of the NICHD Neonatal Network [1-4], the Vermont-Oxford Trials Network [38,39] and the Australia-New Zealand Neonatal Network [40] (although some networks examined all live born infants instead of NICU admissions). Although improvement in survival rates were previously reported among the most preterm groups [1-4,24,38-40], more recent publications report no further improvement in outcomes [41,42], and demonstrate the importance of reporting trends over time.

An advantage in survival for female infants has been reported by previous studies with the greatest difference apparent when stratified by birth weight groups [41-44]. In contrast, when analysed by gestational age, we found a gender difference in survival only for infants born at 24 weeks GA or less. This may reflect the fact that at any given birth weight, female infants tend to be more mature. It has also been previously suggested that the advantage in survival for female infants is related to a more favourable hormonal milieu in the female fetus leading to accelerated lung maturation compared to the male [45-48]. In the present era, it is also possible that the increased use of antenatal steroids and exogenous surfactant has improved overall survival but more notably in male compared to female infants. However, not all gender differences were eliminated and female infants continued to have lower incidences of chronic lung disease and severe intraventricular hemorrhage than male infants. Actuarial survival analysis also showed that female infants had lower mortality rates than male infants during the first 40 days of life, indicating a difference in time of death.

Actuarial analysis adds a further dimension to standard survival data and highlights several points of interest. For all gestational age and birth weight groups the risk of death is greatest during the first few days of life with relatively few deaths occurring after day 7. This emphasises the importance of revising the chances of survival at regular intervals especially during the first few days of life (as in figures 10 and 11). Such updated survival information is useful when informing parents and is necessary for ongoing management decisions. Furthermore, actuarial survival curves are helpful when studying the economic aspects of neonatal intensive care. The majority of deaths occur during the first few days of life and therefore the proportion of health care expenditure on eventual non-survivors is relatively small. With reference to research and health promotion, actuarial data aid the development of treatment strategies. Our data highlights where interventions would have the greatest benefit e.g. early deaths within the first few days and late deaths after the first 28 days. The effects of new interventions on survival patterns could then be monitored. Finally, this report extends the available information on various aspects of low birth weight infants previously reported by the Canadian Neonatal Network [24,26,27,49-58].

One limitation of our survival data is that they only include infants admitted for neonatal intensive care. They do not take into account stillbirths after the onset of preterm labour nor delivery room deaths of live born infants. Therefore, they provide an overestimate of survival chances if used to counsel parents during preterm labour or as a guide to obstetric management. However, as most high risk pregnancies are currently managed in large perinatal centres, with skilled personnel certified in neonatal resuscitation, the majority of infants born at 24 weeks and above are successfully resuscitated and admitted for intensive care, and our results would be highly applicable to them. Discharge policies may affect survival rates but since infants are usually discharged only when they are sufficiently well, it is unlikely that discharge policies will significantly affect our results. For the actuarial survival analysis, survival to 60 days of life was considered. However, deaths may occur even after this time during the post-discharge period and these were not considered in the study.

## Conclusion

Up to date survival rates are essential when evaluating perinatal services. Previously reported effect of gender on overall survival are no longer apparent among infants >24 weeks gestation but there is a gender difference in the time of death. Actuarial survival analysis emphasises the importance of frequently revising predictions for survival in high-risk infants, particularly during the first week of life.

## Abbreviations

BW Birth weight

GA Gestational age

NICU Neonatal intensive care unit

NTISS Neonatal therapeutic intensity scoring system

SNAP-II Score for Neonatal Acute Physiology, Version II

VLBW Very low birth weight

## Competing interests

The author(s) declare that they have no competing interests.

## Authors' contributions

Huw Jones interpreted data and drafted the manuscript. Stella Karuri performed statistical analysis and interpretation. Shoo K Lee was the principal investigator and interpreted the data and drafted the manuscript. Catherine Cronin, Arne Ohlson and Abraham Peliowski and Anne Synnes were site investigators. All these individuals read and approved the final manuscript. The CNN represents all site investigators, and was responsible for organization and administration of the SNAP study, and subsequent data flow.

## Pre-publication history

The pre-publication history for this paper can be accessed here:


